# Different effects of vitamins on hypoxic and normal fetal lungs

**DOI:** 10.1038/s41390-025-03903-7

**Published:** 2025-01-30

**Authors:** Fuat Emre CANPOLAT, Hayriye Gözde KANMAZ KUTMAN

**Affiliations:** Professor of Pediatrics & Neonatology, University of Health Sciences, Ankara Bilkent City Hospital, 06800 Çankaya, ANKARA, Turkey

Some agents administered antenatally may have positive or negative effects on the fetus. Research has been published on many factors known to have very different effects on fetal lung development. This issue is still on the research agenda because it is safer and vitamins have been shown to be effective in experiments. The effects of many drugs such as nutrition, steroids, vitamins, retinoids, growth factors on fetal lungs have been demonstrated in both animal experiments and clinical studies.^1–3^ The main goal of these studies, of course, is to find treatments that will reduce the incidence of bronchopulmonary dysplasia in premature infants. There are data from studies conducted on vitamin A and vitamin D in particular that maternally administered vitamins have positive effects on lung development. Perhaps the most important of these is the study conducted by Greenough et al., but the findings of this team were that antenatal use of vitamins C and E did not have a positive effect on lung outcomes in 2-year-old infants.^3^

**Fig. 1:**
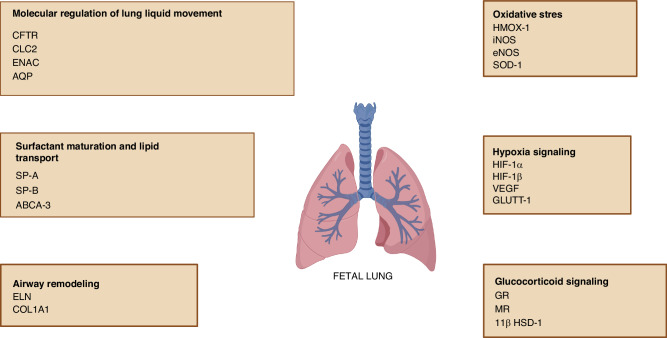
Target genes and proteins regulating oxidative stress, hypoxia signaling, glucocorticoid signaling, lung liquid movement, surfactant maturation and airway remodeling in fetal lung. HMOX-1 Heme oxygenase-1, iNOS Inducible nitric oxide synthase, eNOS Endothelial nitric oxide synthase, SOD-1 Superoxide dismutase enzymes, HIF-1 Hypoxia-inducible factor subunits, VEGF Vascular endothelial growth factor, GLUTT-1 Solute carrier family 2 (facilitated glucose transporter) member 1, GR Glucocorticoid receptor, MR Mineralocorticoid receptor, 11βHSD-1 11β-hydroxysteroid dehydrogenase enzyme -1, CFTR Cystic fibrosis transmembrane conductance regulator, CLC2 Chloride channel voltage-sensitive 2 channel, ENAC Epithelial sodium channel subunits, AQP Aquaporins, SP-A, SP-B Surfactant proteins, ABCA-3 ATP-binding cassette, sub-family A (ABC1), member 3, ELN Elastin, COL1A1 Collagen type 1 alpha 1.

When we look at other studies, especially those with vitamins A and C, it may have positive effects on the fetal lungs.^4^ Although most of the research consists of animal experiments, the mechanisms of action, receptors, possible gene locations and intracellular and extracellular signaling cascades have been clearly explained and demonstrated (Fig. 1).^5,6^ According to this paper’s results, the authors observed that, maternal vitamin C treatment increased fetal lung gene expression of the antioxidant enzymes, hypoxia signaling genes, genes regulating sodium movement, surfactant maturation, and airway remodeling. Additionally they founded that there was no effect of maternal vitamin C treatment on the expression of protein markers evaluated or on the number of surfactant protein-producing cells in fetal lung tissue.^5^

The same team that did this study planned and presented this latest publication. The main purpose of the study was to see and compare the effects of vitamin C on a normally developing normoxic lung and a hypoxic lung that is not normally developing, given that these effects are known. So that the most important difference that distinguishes this study from others is that the lungs in one group were exposed to hypoxia in intrauterine life and the effect of vitamin C on the lungs of a fetus has growth restriction was investigated.^7^ According to this results, authors showed a differential effect of antenatal Vitamin C treatment on regulation of genes involved in maturation of surfactant, sodium movement and hypoxia signaling. Limited responsiveness to antenatal Vitamin C exposure in the lung of the hypoxic fetus, compared to responsiveness to antenatal Vitamin C in the normoxic fetus, suggests a maximal upregulation of the molecular signaling pathways in response to the chronic hypoxic insult alone. Although this result may be predictable or foreseeable, it has been demonstrated very well and has become valuable data because the effects of intrauterine hypoxia on both gene, surfactant and sodium mechanisms have been revealed in great detail.

As a result, we can conclude that when studying the effects on the fetal lung, the responses of normal and hypoxic lungs will be different, that many molecules that can affect the lung, including vitamins, are worth studying, and that these lung studies should continue and that experimental and clinical research should be given strength and speed to prevent bronchopulmonary dysplasia in premature babies.
